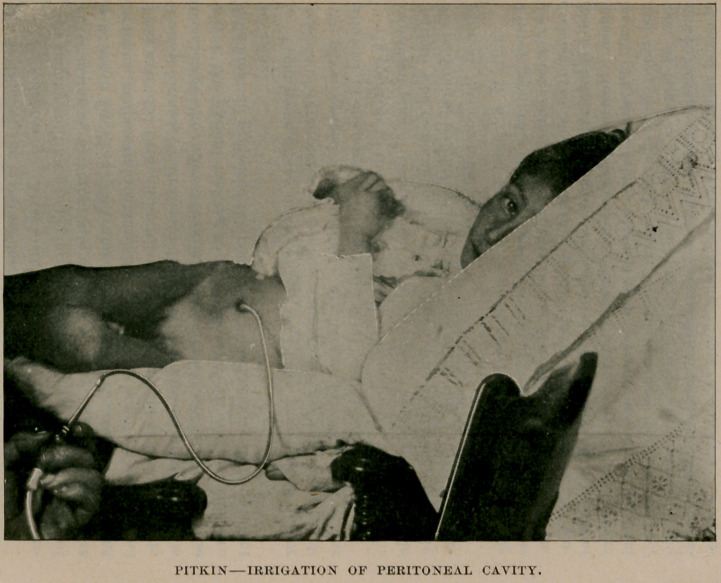# Irrigation of the Peritoneal Cavity

**Published:** 1896-02

**Authors:** John T. Pitkin

**Affiliations:** Buffalo, N. Y.; 206 Connecticut Street


### IRRIGATION OF THE PERITONEAL CAVITY.

By JOHN T. PITKIN, M. D., Buffalo, N. Y.

WHENEVER the general peritoneal sac becomes the receptacle of a purulent discharge from (*a*) an hepatic abscess, (*b*) a suppurative mesenteric gland, (*c*) a pyosalpinx not radically removed, (*d*) pyothorax via the diaphragm, (*e*) suppurative peritonitis, local or diffuse, I would earnestly advocate reopening the umbilical fenestrum, the insertion of a soft rubber drainage-tube therein, allow of its free escape therefrom, followed by repeated irrigation *ad libitum* with a warm, sterilised, normal saline and antiseptic solution.

As to the desirability of thus thoroughly cleansing the peritoneal cavity for the relief of pyo-peritoneum in these days of modern antiseptic surgery, it seems to me, no progressive medical mind will question.

That the navel should be elected the point of entrance, let us look to anatomy, physiology and pathology as our indices.

*Anatomically.*—The navel presents the thinnest point in the abdominal parietes, consisting of varying amounts of cicatricial

PITKIN—IRRIGATION OF PERITONEAL CAVITY.

tissue, the center of which is perforated by the whip-cord like remnants of the umbilical arteries, vein and urachus. Obviously the strength of the abdominal walls at the omphalos must vary extremely in different subjects. In many, especially the young, before the scar-flesh has become strengthened by contraction, it is the point of least resistance. It is without adipose or muscular tissue, and for surgical purposes practically nonvascular.

*Physiologically.*—In intrauterine life the navel is the portal of communication between the fetus and the outer world, through the medium of the maternal blood.

*Pathologically.*—After birth the navel, not infrequently, in a limited manner continues to perform, or reassumes in the elimination of effete material, its embryonic usefulness—*e. g.*, I have seen in the practice of Dr. M. a child who voided his urine through a patulous urachus, and several instances are recorded where fecal matter has been extruded through an intestinal diverticulum at this aperture. I am informed by Professor Henry R. Hopkins of a patient in his clientèlage who suffered from an hepatic abscess which pointed at the navel, the patient making a good recovery. In this case adhesive inflammation protected the general peritoneal cavity from purulent invasion.

From some of my other colleagues I have been able to gather reports of three cases of general suppurative peritonitis in which the navel opened spontaneously, discharged for several days, was allowed to close again, followed by fatal consequences.

In contrast to the untoward result obtained from a let-alone or expectant plan of treatment, I would narrate the history of a little patient who came under my observation, was treated aggressively by peritoneal irrigation, and which has made that subject the purpose of this communication.

Julia M. K., aged 4, German descent; previous personal and family history good; only child. September 29th, after excessive gastronomic indulgence, enteritis ensued with twenty to thirty movements per diem. October 10th, stools infrequent, general peritonitis developed. November 1st, inflammatory processes subsided, patient allowed freedom of house. November 6th, small bunch, size of hickory nut, protrudes from navel. Physician being undecided as to its nature was dismissed from the case. November 9th, bunch has continuously increased in size, now as large as a lemon; constipation alternates with diarrhea. Second physician called. Diagnosis—from location, serous covering, crepitation and reducibility—umbilical hernia. Truss recommended. November 10th, rupture of bunch took place, considerable fetid matter liberated. November 18th, purulent discharge decreasing, opening at navel growing small, obstipation and emesis pronounced. November 19th, 20th, 21st and 22d, complete obstruction, all food rejected by stomach, emaciation marked, medicines of no avail; prognosis of physician, child must die. November 22d, 10.30 P. M., as a last resort the writer was summoned to the patient's bedside. The little face was drawn and pinched, pulse hardly perceptible at the wrist. For five days vomiting had been unabatable, nothing had passed the bowels, urine very scanty and high colored—nearly suppressed—emaciation was extreme, little more than skin and bone remained of an interesting child.

Her abdomen was greatly distended and tympanitic; most marked over the small intestines, dulness in hypogastrium. Diagnosis, pyo-peritoneum and obstruction to lower small intestines by pressure and adhesions. Treatment: reopened navel, liberated large quantity of foul matter. Inserted soft rubber drainage-tube, through which liberal injections of warm water, sterilised by boiling and rendered alkaline, and antiseptic by the addition of Seiler's tablets, six to the pint. Similar injections were administered per rectum, peptonised food by mouth and rectum. Peritoneal irrigation was performed daily for over a week, then with longer intervals until the wash water returned perfectly clear. The navicular opening was then allowed to close, the patient making an uninterrupted recovery. By the process employed all foreign matter was removed from the peritoneum, its cavity cleansed and the adherent surfaces separated from each other by hydrostatic pressure. (See Fig., p. 549.)

Are we not led to conclude that the navel is a semi-normal passage, a sealed abdominal os, the reopening of which may be frequently indicated and accomplishable, either by natural forces or the surgeon's knife, with the danger of shock and collapse reduced to a minimum, and that thorough repeated aseptic irrigation of the peritoneal cavity may be demanded as a life-saving measure whenever that structure has been invaded by bacteria or their products?

206 CONNECTICUT STREET.

## Figures and Tables

**Figure f1:**